# Comparison of clinical and immunological findings in gnotobiotic piglets infected with *Escherichia coli* O104:H4 outbreak strain and EHEC O157:H7

**DOI:** 10.1186/s13099-017-0179-8

**Published:** 2017-05-25

**Authors:** Bettina Wöchtl, Florian Gunzer, Wilhelm Gerner, Hagen Gasse, Michaela Koch, Zoltán Bagó, Martin Ganter, Herbert Weissenböck, Nora Dinhopl, Sina M. Coldewey, Alexandra von Altrock, Karl-Heinz Waldmann, Armin Saalmüller, Kurt Zimmermann, Jörg Steinmann, Jan Kehrmann, Ludger Klein-Hitpass, Jochen Blom, Ralf Ehricht, Ines Engelmann, Isabel Hennig-Pauka

**Affiliations:** 1 University Clinic for Swine, Department for Farm Animals and Veterinary Public Health, University of Veterinary Medicine Vienna, Veterinärplatz 1, 1220 Vienna, Austria; 2 Institute of Medical Microbiology and Hygiene, Faculty of Medicine Carl Gustav Carus, TU Dresden, Fetscherstrasse 74, 01307 Dresden, Germany; 3 Institute of Immunology, Department of Pathobiology, University of Veterinary Medicine Vienna, Veterinärplatz 1, 1220 Vienna, Austria; 4 Institute of Anatomy, University of Veterinary Medicine Hannover, Foundation, Bischofsholer Damm 15, 30173 Hannover, Germany; 5 Institute for Veterinary Disease Control Mödling, Austrian Agency for Health and Food Safety, Robert-Koch-Gasse 17, 2340 Mödling, Austria; 6 Clinic for Swine, Small Ruminants, Forensic Medicine and Ambulatory Service, University of Veterinary Medicine Hannover, Foundation, Bischofsholer Damm 15, 30173 Hannover, Germany; 7 Institute for Pathology and Forensic Veterinary Medicine, Department of Pathobiology, University of Veterinary Medicine Vienna, Veterinärplatz 1, 1220 Vienna, Austria; 8 Department of Anaesthesiology and Intensive Care Medicine, University Hospital Jena, Am Klinikum 1, 07747 Jena, Germany; 9 Centre for Innovation Competence (ZIK) Septomics, University Hospital Jena, Albert-Einstein-Strasse 10, 07745 Jena, Germany; 10 SymbioPharm GmbH, Auf den Lüppen 10, 35745 Herborn, Germany; 11 Institute of Medical Microbiology, University Hospital Essen, University of Duisburg-Essen, Hufelandstraße 55, 45147 Essen, Germany; 12 Institute of Cell Biology, Medical Faculty, University Hospital Essen, University of Duisburg-Essen, Hufelandstraße 55, 45147 Essen, Germany; 13 Bioinformatics and Systems Biology, Justus-Liebig-University Giessen, Heinrich-Buff-Ring 58, 35392 Gießen, Germany; 14 Alere Technologies GmbH, Löbstedter Straße 103-105, 07749 Jena, Germany

**Keywords:** Swine, Gnotobiotic piglets, Enteroaggregative *E. coli*, Enterohaemmorrhagic *E. coli*, Shiga toxin, Haemolytic uraemic syndrome (HUS), *E. coli* O104:H4, *E. coli* O157:H7

## Abstract

**Background:**

Shiga toxin (Stx) producing *Escherichia coli* (*E. coli*) (STEC) is the most frequent cause of diarrhoea-positive haemolytic uraemic syndrome (D + HUS) in humans. In 2011, a huge outbreak with an STEC O104:H4 strain in Germany highlighted the limited possibilities for causative treatment of this syndrome. The responsible STEC strain was found to combine Stx production with adherence mechanisms normally found in enteroaggregative *E. coli* (EAEC). Pathotypes of *E. coli* evolve and can exhibit different adhesion mechanisms. It has been shown previously that neonatal gnotobiotic piglets are susceptible for infection with STEC, such as STEC O157:H7 as well as for EAEC, which are considered to be the phylogenetic origin of *E. coli* O104:H4. This study was designed to characterise the host response to infection with the STEC O104:H4 outbreak strain in comparison to an STEC O157:H7 isolate by evaluating clinical parameters (scoring) and markers of organ dysfunction (biochemistry), as well as immunological (flow cytometry, assessment of cytokines/chemokines and acute phase proteins) and histological alterations (light- and electron microscopy) in a gnotobiotic piglet model of haemolytic uraemic syndrome.

**Results:**

We observed severe clinical symptoms, such as diarrhoea, dehydration and neurological disorders as well as attaching-and-effacing lesions (A/E) in the colon in STEC O157:H7 infected piglets. In contrast, STEC O104:H4 challenged animals exhibited only mild clinical symptoms including diarrhoea and dehydration and HUS-specific/severe histopathological, haematological and biochemical alterations were only inconsistently presented by individual piglets. A specific adherence phenotype of STEC O104:H4 could not be observed. Flow cytometric analyses of lymphocytes derived from infected animals revealed an increase of natural killer cells (NK cells) during the course of infection revealing a potential role of this subset in the anti-bacterial activity in STEC disease.

**Conclusions:**

Unexpectedly, *E. coli* O104:H4 infection caused only mild symptoms and minor changes in histology and blood parameters in piglets. Outcome of the infection trial does not reflect *E.* *coli* O104:H4 associated human disease as observed during the outbreak in 2011. The potential role of cells of the innate immune system for STEC related disease pathogenesis should be further elucidated.

## Background

Shiga toxin (Stx) producing *Escherichia coli* (*E. coli*) (STEC) is a frequent cause of D + HUS in humans. HUS is characterized by acute renal failure, haemolytic anaemia and thrombocytopenia [1]. In 2011 an outbreak of STEC O104:H4 associated disease occurred in Germany. In total, 3816 clinical cases and the death of 54 persons were reported between May 1st and July 4th. In contrast to the epidemiological course of disease in outbreaks due to classical EHEC infections, the percentage of patients suffering from HUS was high (22%) and most of the HUS patients were female adults and not children [2].

Attaching and effacing (A/E) lesions in the gut are a hallmark for EHEC infection. The genes involved in A/E mechanisms are located on the locus of enterocyte effacement (LEE) that contains, amongst others, the *eae* gene encoding intimin. As various alternative adhesion mechanisms have been described in STEC so far, the terms STEC and EHEC should not be used synonymously [3]. All STEC including EHEC have in common that they produce one or more Stxs in the intestine [3]. Globotriaosylceramide (Gb3)-dependent internalisation of Stxs into sensitive cells has been demonstrated [4]. Previously, an alternative mechanism could be shown. Stx produced by EHEC O157:H7 [5] and *E. coli* O104:H4 [6] can be released by outer membrane vesicles (OMV). Subsequent, OMVs and their contents can be internalised to human intestinal epithelial cells (IEC) [6].

The outbreak strain of 2011 produced Stx2a, extended-spectrum beta-lactamases (ESBL) and exhibited the adherence mechanism of EAEC [2]. *E. coli* O104:H4 is considered an emerging pathogen endowed with virulence factors from different strains. Up to now, a conclusive explanation for the severity of the outbreak and the clinical and epidemiological differences compared to other and better known STEC strains of enteropathogenic *E.* *coli* (EPEC) origin is lacking. It was previously hypothesised that the different adherence mechanisms of *E. coli* O104:H4 may be the reason for the severity of the outbreak [7–9]. Another explanation may be that specific virulence factors of the strain facilitate disruption of the epithelial barrier and Stx-transfer to circulation [9]. Amongst others, three serine protease autotransporters produced by *E.* *coli* O104:H4 may contribute to an increase in Stx intake [10].

Understanding pathogenesis of HUS is the prerequisite for the development of new preventive and therapeutic strategies for this syndrome. While many bacterial characteristics have been elucidated so far, knowledge about the hosts innate and adaptive immune reactions as well as genetically determined susceptibility and co-factors for disease is fragmentary. Recently, the decisive role of natural killer T cells (NKT) for Stx2-induced pathology was shown in mice [11]. Stx2-binding to Gb3 led to an aberrant CD1d-mediated NKT cell activation in podocytes and glomerular endothelial cells expressing the CD1d molecule. It was assumed that Stx2-induced co-stimulatory molecules in renal cells led to NKT cell activation [11].

Various animal models are used to investigate aspects of pathogenesis in STEC associated disease [12–16]. Gnotobiotic piglets infected with Stx-producing *E. coli* O157:H7 and *E. coli* O26:H11 developed clinical and pathological features of HUS, which qualified the model for reproduction of human STEC-related disease [15]. Neonatal gnotobiotic piglets were also successfully used for EAEC infection experiments [17]. Based on these former experiences, the gnotobiotic piglet model was assessed for parallel infection experiments with *E. coli* O104:H4 and EHEC O157:H7. An infection model described previously [15] was adapted with only slight modifications.

The aim of this study was to compare clinical outcome and underlying pathological mechanisms of infection with LEE-negative *E. coli* O104:H4 and LEE-positive *E. coli* O157:H7 employing a gnotobiotic piglet model of HUS by using oral infection with these strains. Specifically, we assessed over the course of the experiment haematological and biochemical parameters indicating STEC-related disease in humans and we compared the colonisation characteristics of both strains in the porcine intestine by electron microscopy and bacteriological examination. Furthermore, we tested in vivo Stx production in the intestine by performing Stx ELISA from stool samples. Immunological analyses, such as phenotyping of peripheral blood mononuclear cells (PBMCs) and lymphocytes from mesenterial and ileocaecal lymph nodes by flow cytometry were executed to address the open question, which cell populations may be of importance in STEC disease. Circulating cytokines/chemokines were determined by using fluorescent microsphere immunoassays (FMIA).

## Methods

### Delivery and infection experiments in gnotobiotic piglets

Gnotobiotic piglets were derived from four German Landrace sows by Caesarian section in three consecutive trials as described previously [15]. During the trial, sterility was tested by regular bacteriological examination of swabs from the animals and the isolators at the end of the experiment.

In total, 55 piglets were delivered from the four sows. For this study, data of 13 piglets belonging to three different experimental groups were included into evaluation: control group (n = 3) and infection groups *E. coli* O104:H4 (n = 6) and *E. coli* O157:H7 (n = 4), respectively. All other piglets were of low vitality, did not stay sterile or had been used for other experiments.

Piglets were infected orally 12 h after birth with 5 ml bacterial suspension using a curved olive headed probe outreaching the root of tongue. The control group received sterile sodium chloride (0.9% NaCl) (Table 1).

**Table 1:** Infective dose administered, bacteriological and clinical findings in individual animals

Group animal number	Infective dose (CFUs)	CFUs/g stool day 4 p.i.	CFUs/g stool day 5–6 p.i.	OD Stx ELISA day 5–6 p.i.	CFUs/g stool day 9–12 p.i.	OD Stx ELISA day 9–12 p.i.	Average clinical score (animal/group)	Clinical signs, assessed at least once	Day of death
Negative									
1	Mock	Sterile	Sterile	n.a.	Sterile	0.009 (N)	0.00	None	10
2	Mock	Sterile	Sterile	0.025 (N)	Sterile	0.012 (N)	0.11	Reduced appetite	9
3	Mock	Sterile	Sterile	0.020 (N)			0.00	None	6^a^
							Ø 0.04		
*E. coli* O104:H4									
4	1.00 × 10^9^	1.22 × 10^10^	2.81 × 10^10^	n.a.	8.60 × 10^8^	4.538 (P)	0.53	Reduced appetite, dehydration	10
5	1.00 × 10^9^	8.17 × 10^9^	6.88 × 10^10^	n.a.	2.62 × 10^9^	3.993 (P)	0.51	Reduced appetite, dehydration, mild central nerval signs	10
6	2.19 × 10^8^	2.13 × 10^10^	3.38 × 10^7^	n.a.	Sterile	2.746 (P)	0.46	Reduced appetite, dehydration	10
7	2.19 × 10^8^	1.18 × 10^7^	2.10 × 10^9^	n.a.	Sterile	3.492 (P)	0.52	Reduced appetite, mild central nerval signs	9
8	2.19 × 10^8^	3.13 × 10^9^	1.63 × 10^9^	4.122 (P)			0.07	Reduced appetite	6^a^
9	2.19 × 10^8^	5.58 × 10^9^	1.11 × 10^10^	2.833 (P)	4.65 × 10^7^	4.561 (P)	0.11	Reduced appetite, dehydration	12
							Ø 0.37		
*E. coli* O157:H7									
10	2.20 × 10^8^	5.43 × 10^9^		.			0.74	Severe neurological signs	4^b^
11	2.20 × 10^8^	1.02 × 10^10^	8.35 × 10^9^	n.a.	5.95 × 10^9^	4.599 (P)	0.67	Mild central nerval signs, dehydration	11
12	2.08 × 10^8^	1.95 × 10^7^	2.50 × 10^7^	4.358 (P)			0.30	Severe neurological signs	5^b^
13	2.08 × 10^8^	1.30 × 10^9^	1.37 × 10^8^	4.527 (P)			0.50	Dehydration	5^b^
							Ø 0.55		

Interpretation Shiga toxin ELISA: optical density (OD) < 0.070 = negative; OD > 0.070/<0.100 = indefinite, ODs > 0.100 = positive

*n.a*. not assessed, *N* negative, *P* positive

^a^Euthanized early due to organisational reasons

^b^Euthanized early due to severe clinical symptoms

### Bacterial strains

*Escherichia coli* O157:H7 strain 86-24 originates from a meat associated EHEC outbreak in Walla Walla WA [18]. This intimin positive strain has an A/E phenotype, synthesizes Stx2 and is able to cause a D + HUS like disease in gnotobiotic piglets [15].

*Escherichia coli* O104:H4 strain e2975 is a stool isolate obtained from a 16-year-old male patient, who was admitted to the University Hospital Essen, Essen, Germany with bloody diarrhoea during the 2011 EHEC outbreak in Germany. In the further course the patient developed HUS and also exhibited seizures. The strain was geno-serotyped using the *E. coli* PanType AS-2 DNA oligonucleotide microarray [19] from Alere Technologies (Jena, Germany). Stx subtyping was performed with the ShigaToxType AS-2 from Alere Technologies [20]. Furthermore, the complete genome of the strain was determined on an Illumina HiSeq sequencing system (Illumina, San Diego, CA, USA). Like all isolates of that outbreak, it bears the serotype O104:H4 and is a chimera between EHEC and EAEC. It displays a blended phenotype with characteristics of both *E. coli* pathotypes [7, 21, 22]. The strain produces Stx2 variant a, lacks intimin and enterohemolysin, is positive for *iha* encoding an adherence-conferring protein homologous to IrgA [23] and shows an ESBL resistance pattern due to plasmid encoded TEM-1 and CTX-M-M15 β-lactamases. It also possesses *mchB* coding for the microcin H47 activity protein [24].

*Escherichia coli* cultures for oral infection were prepared in advance and stored in 0.9% NaCl at room temperature. Briefly, 4 ml overnight culture of the respective bacterial strain were grown in LB broth (LBB; 10 g/l tryptone, 10 g/l NaCl, 5 g/l yeast extract) and inoculated at an optical density OD_600_ of 0.06 in 100 ml LBB the following day. When a specific OD_600_ was reached, individually determined for each strain according to growth curves, bacteria were harvested by centrifugation (50 ml tubes, 4000×*g* at 4 °C for 10 min). The supernatant was discarded and the pellet was washed once with phosphate buffered saline (PBS; 1.4 mM NaCl, 116 mM NaH_2_PO_4_, 18 mM KH_2_PO_4_) to remove any free Stx or degradation products. Cultures were diluted in 0.9% NaCl and colony forming units (CFU) were determined by plating and counting serial dilutions. Bacterial suspensions were stored at room temperature until further use. Piglets were orally infected with 5 ml liquid culture with doses ranging from 2.08 × 10^8^ to 2.20 × 10^8^ CFU for *E. coli* O157:H7 and 2.19 × 10^8^ to 1.00 × 10^9^ CFU for the *E. coli* O104:H4 outbreak strain.

### Clinical and laboratory diagnostic monitoring

A visual inspection of animals was performed every 2 h and clinical scores were recorded every 4 h. Appetite, neurological signs and status of hydration were separately assessed as physiological (score 0), mild (score 1) and severe (2) and scores were added to calculate a total score. A total clinical score for each individual was generated by dividing sum of all scores through number of examination time points.

If clinical scores were assessed to be ≥2 at two consecutive examination times the respective pig was euthanised due to animal welfare reasons. The experiment was finalised on days 9–12 after infection by euthanasia of piglets (Table 1). Immediately after death, pigs were removed from the isolator, weighed and dissected under a S2 laminar air flow workbench.

Blood was sampled from the *Vena cava cranialis* immediately prior to infection (day 0), on day 6 after infection and prior to euthanasia at the end of the experiment. Urine samples were taken on day 6 after infection and prior to euthanasia. Faeces samples were collected prior to infection, on days 4 and 6 and after euthanasia.

Red and white blood cells as well as clinical chemistry parameters were determined according to routine diagnostic methods described elsewhere with slight modifications [25]. Acute phase proteins haptoglobin (Tridelta Phase Haptoglobin Assay, Tridelta Development Limited, Maynooth, Ireland), C-reactive protein (Phase Porcine CRP Assay, Tridelta Development Limited) and lipopolysaccharide-binding proteins (LBP ELISA, Hycult Biotech, Uden, Netherlands) were determined according to the manufacturer’s instructions of used detection kits.

Urine was collected on day 6 and day of death and processed for routine diagnostics, including refractometry, urine chemistry and testing by Combur 9 test strips (Roche Diagnostics, Mannheim, Germany). In sediments the amount of bacteria, leukocytes, superficial squamous epithelial cells, round epithelial cells and deeper transitional epithelial cells as well as crystals were assessed by microscopical inspection. The glomerular filtration rate (GFR) and the fractional excretions (FE) of water and Na were calculated based on the endogenous marker creatinine, which is exclusively eliminated by glomerular filtration [26].

During necropsy organ tissue samples from all parenchymatous organs, gastro intestinal tract and brain were collected for histological examination. Tissue from *Colon ascendens*, cerebellum and kidney were sampled for electron microscopy (EM).

Faecal samples were obtained on day 4, 6 and day of necropsy. A 1:10 starting solution was prepared by dissolving 100 mg faeces in 900 µl PBS and was then diluted further up to 10^−8^. 10 µl of each serial dilution were spotted clockwise on Müller-Hinton agar (Oxoid, Thermo Fisher Scientific, Wesel, Germany), sorbitol MacConkey agar (SMAC, Oxoid) and CHROMagar ESBL (MAST DIAGNOSTICA, Reinfeld, Germany) in parallel to obtain a general overview of the bacterial load in the fecal samples. For detailed analysis 50 µl of bacterial suspension were spread on an entire agar plate.

After incubation for 18 h at 37 °C, CFU/g faeces were calculated from bacterial counts obtained with appropriate agar plates of the dilution series. Semiquantitative determination of Stx2 in faecal samples was carried out using the ProSpecT Shiga Toxin *E. coli* (STEC) Microplate Assay (Oxoid) according to the manufacturer´s instructions.

### Light and electron microscopy

All tissue samples were fixed and stained according to routine histological methods as described previously [27]. After immediate fixation in 10% buffered formalin samples were embedded in paraffin wax. Paraffin sections (6 µm) were either stained with hematoxylin–eosin (HE, hemalaun after Delafield) or with Masson´s trichrome stain for routine histological examination.

Multiple small 1 mm^3^ tissue blocks from the renal cortex, the colonic mucosa, and the cerebellum were sampled for electron microscopy and fixed in 5% glutaraldehyde in phosphate-buffered saline (PBS). After an adequate fixation time of at least 24 h samples were washed in PBS, postfixed for 90 min in 1% osmium tetroxide in double-distilled water, subsequently rinsed in 30, 50 and 70% ethanol. In a next step tissue was infiltrated with uranylacetate and phosphoric tungstic acid within 2 h. After subsequent 60-min-treatments with 90% ethanol, 100% ethanol and propylene oxid, tissue was stored in epon (Epon 812, Fluka, Buchs, Switzerland): propylene oxid in a 1:1 mixture at 4 °C overnight. The next day samples were infiltrated with pure epon for 1 h and poured in silicon forms, followed by two 24-h-polymerization steps at 35 and 45 °C and one 60-h-polymerization step at 60 °C. Toluidine blue stained thin sections (1.5 μm) from each tissue block were inspected for selection of areas for EM ultrathin sectioning. Ultrathin sections were cut on a Leica Ultramicrotome (Leica Ultracut S, Vienna, Austria) and stained with uranyl acetate (Sigma-Aldrich, Vienna, Austria) and lead citrate (Merck, Darmstadt, Germany). Ultrathin sections were examined with a Zeiss TEM 900 electron microscope (Carl Zeiss, Oberkochen, Germany) operated at 50 kV.

### Isolation and phenotyping of lymphocytes and myeloid cells

In accordance with a previously established protocol [28], PBMCs were isolated from heparinized blood by density gradient centrifugation using lymphocyte separation medium (Pancoll human, density 1.077 g/ml, PAN Biotech, Aidenbach, Germany), which had been adjusted by PBS (without Ca^2+^ and Mg^2+^, PAN Biotech) dilution to a density of 1.075 g/ml. Mesenterial and ileocaecal lymph nodes were dissected during necropsy from surrounding tissue, pooled for each individual animal and further processed as described elsewhere [29].

Isolated cells from blood and lymph nodes were suspended in PBS supplemented with 10% porcine plasma (in-house preparation). Cell labelling was performed in 96-well round-bottom microtiter plates (Greiner Bio One, Frickenhausen, Germany) with 2 to 5 × 10^5^ cells per sample. Antibodies and second-step reagents used for labelling are listed in Table 2. Prior to use, optimal working dilutions for all antibodies and second-step reagents had been determined in titration experiments. Mastermixes of antibodies were prepared freshly before use. After addition of primary antibodies samples were incubated for 20 min at 4 °C, followed by two washing steps in 200 μl of PBS. A plate shaker was used for resuspension of cells after washing steps. Secondary reagents were added and also incubated for 20 min at 4 °C, followed by two washings steps with PBS + 10% porcine plasma. For discrimination of dead cells Fixable Near-IR Dead Cell Stain Kit (Invitrogen, Carlsbad, CA, USA) was used during the second incubation according to manufacturer’s instructions. In a further incubation step fixation and permeabilization was performed with the BD Cytofix/Cytoperm kit (BD Biosciences, San Jose, CA, USA) according to the manufacturer`s instructions. This allowed the staining of the intracellular epitope recognized by the anti-CD79α antibody (Table 2).

**Table 2:** Antibody panels used for immunophenotyping by flow cytometry

Cell population addressed	Antigen	Clone	Isotype	Fluorochrome	Labelling strategy	Source of primary Ab
Myeloid cells, B cells and CD4^+^ T cells	CD3	PPT3	IgG1	BV 421^a^	Biotin–streptavidin	In-house
	CD79α	HM57	IgG1	PE	Direct conjugation	Dako
	CD172α	74-22-15	IgG1	Alexa Fluor 647^b^	Direct conjugation	In-house
	CD4	74-12-4	IgG2b	Alexa 488^c^	Secondary antibody	In-house
T cells and NK-cell subpopulations	CD3	PPT3	IgG1	BV 421^a^	Biotin–streptavidin	In-house
	CD4	74-12-4	IgG2b	Alexa Fluor 488^c^	Secondary antibody	In-house
	CD8α	11/295/33	IgG2a	PE^d^	Secondary antibody	In-house
	NKp46	VIV-KM1	IgG1	Alexa Fluor 647^b^	Direct conjugation	In-house
γδ T-cell subpopulations	TCR-γδ	PPT16	IgG2b	BV421^a^	Biotin–streptavidin	In-house
	CD8α	11/295/33	IgG2a	Alexa Fluor 647^b^	Direct conjugation	In-house
	CD27	b30c7	IgG1	PE^e^	Secondary antibody	In-house
	CD2	MSA4	IgG2a	Alexa Fluor 488^f^	Direct conjugation	In-house

^a^Brilliant Violet 421 streptavidin, BioLegend, San Diego, CA, USA

^b^Alexa Fluor 647 Protein Labelling Kit, Invitrogen, Carlsbad, CA, USA

^c^Goat anti-Mouse IgG2b-Alexa Fluor 488, Invitrogen, Carlsbad, CA, USA

^d^Goat anti-Mouse IgG2a-PE, Southern Biotech, Birmingham, AL, USA

^e^Goat anti-Mouse IgG1-PE, Southern Biotech, Birmingham, AL, USA

^f^Alexa Fluor 488 Protein Labelling Kit, Invitrogen, Carlsbad, CA, USA

For each fluorochrome single stain samples were prepared at the time when the staining protocol was established and used for automatic calculation of compensation by FACSDiva software Version 6.1.3 (BD Biosciences).

**Fig. 1:**
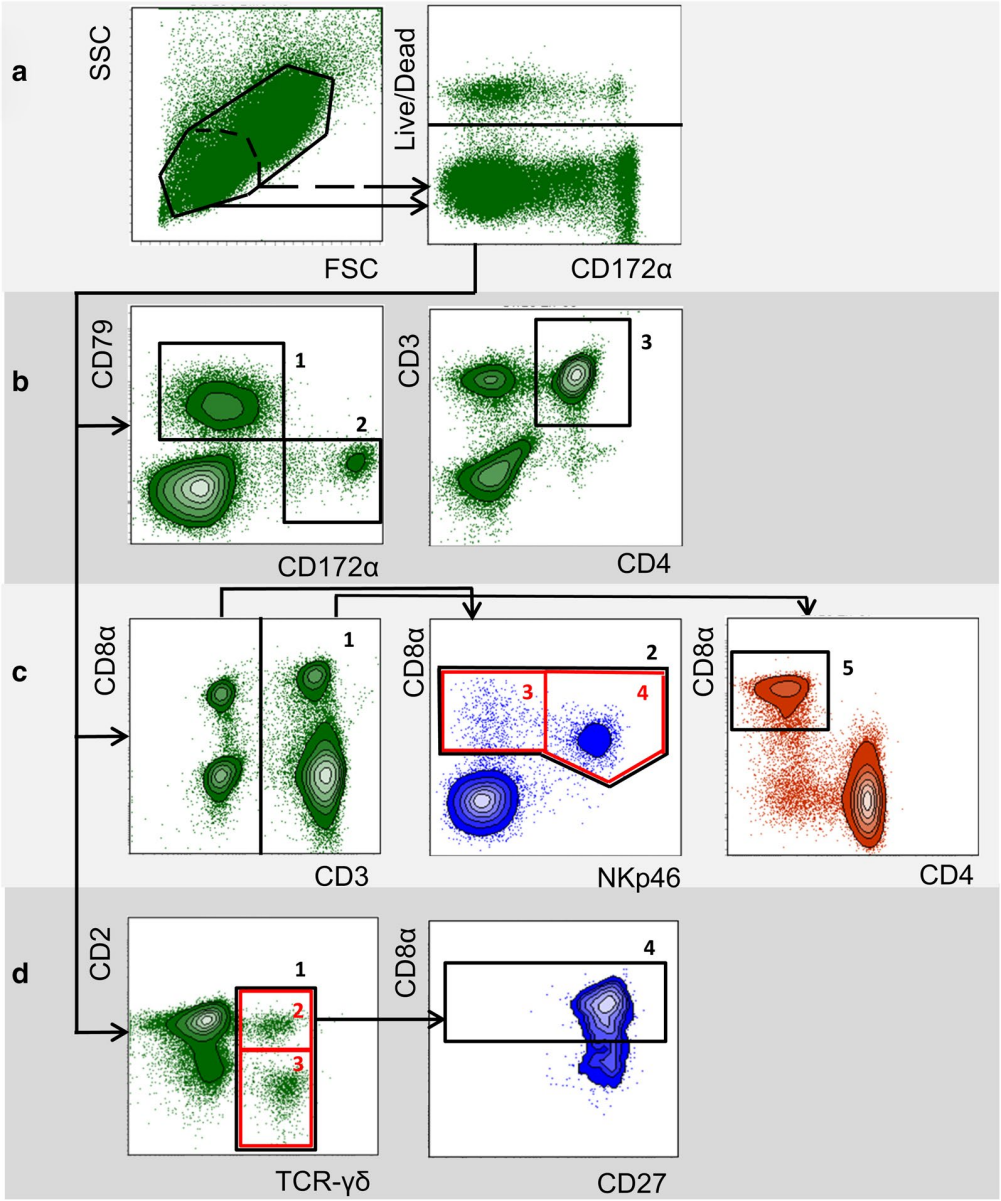
Gating strategy in flow cytometry for identification of myeloid cells and lymphocyte subpopulations. Representative data from one animal is shown. **a** Lymphocytes (*scattered line*) or lymphocytes and myeloid cells together (*solid line*) were gated according to forward scatter/side scatter properties (FSC/SSC). Dead cells were excluded in a consecutive gate. Live cells were further analysed by three different *panels* of antibodies (**b**–**d**, see also Table 2), used to identify various immune cell subpopulations. **b** *Gate 1* CD79α^+^ cells (B cells); *gate 2* CD172 α^+^ cells (myeloid cells); *gate 3* CD3^+^CD4^+^ cells (CD4 T cells). **c** *Gate 1* CD3^+^ cells (total T cells); *gate 2* CD3^−^CD8α^+^ cells (total NK cells); *gate 3* (*red rectangle*): CD3^−^CD8α^+^NKp46^−^ cells (NK-cell subpopulation); *gate 4* (*red rectangle*): CD3^−^CD8α^+^NKp46^+^ cells (NK-cell subpopulation); *gate 5* CD3^+^CD4^−^CD8 α^+^ (cytolytic T cells). **d** *Gate 1* TCR-γδ^+^ cells (total γδ T cells); *gate 2* (*red rectangle*): CD2^+^TCR-γδ^+^ cells (γδ T-cell subpopulation); *gate 3* (*red rectangle*): CD2^−^TCR-γδ^+^ cells (γδ T-cell subpopulation); *gate 4* CD2^+^CD8α^+^CD27^+/−^TCR-γδ^+^ cells (γδ T-cell subpopulation)

Samples were analysed on a BD FACSCanto II flow cytometer equipped with a high-throughput sampler and three lasers (405, 488 and 633 nm, BD Biosciences) and subsequently analysed by FACSDiva software (Fig. 1). In PBMC numbers of analysed cells ranged from 12,000 to 132,000 and in lymph nodes cells from 14,000 and 72,000. Total numbers of cell subpopulations were calculated based on white blood cell counts and percentages of cells resulting from flow cytometry.

### Cytokine testing by FMIA

The following circulating cytokines/chemokines were determined by applying FMIA on serum samples as described elsewhere [30]: interleukin (IL)-1-beta (IL1β), IL4, IL8, IL10, IL12, interferon gamma (IFNγ) and chemokine ligand (CCL) 2. Used antibodies and standards are listed in Table 3.

**Table 3:** Standards, capture and detection antibodies used by FMIA

Cytokine (bead region)	Standard	Capture Ab	Detection Ab
IL1β (26)	DY681, part 681-PI^a^	MAB6811^a^	BAF 681^a^
IL4 (34)	CSC1283, part 5S.128.10^b^	CSC1283, part 5S.128.09^b^	ASC0849^b^
IL8 (27)	RP0109S-005^c^	MCA1660^d^	MAB5351^a^
IL12 (36)	912-PL^a^	MAB9121^a^	BAM9122^a^
CCL2 (53)	RP0017S-005^c^	Anti-poCCL2 clone 5-2^e^	Anti-poCCL2 clone 18-1^e^
IFNγ (43)	PPP022^d^	MP700^b^	MP701B^b^
IL10 (28)	CSC0103, part SD064^b^	ASC0104^b^	ASC9109^b^

^a^R&D Systems, Minneapolis, MN, USA

^b^Thermo Fisher Scientific, Waltham, MA USA

^c^Kingfisher Biotech, Biomol, Hamburg, Germany

^d^Bio Rad, Hercules, CA, USA

^e^Lunney lab, Beltsville, MD, USA

### Statistical evaluation

For statistical analysis laboratory diagnostic findings from samples taken between day 4 and 6 after infection were compared between the groups by Mann–Whitney U test (SPSS Statistics for Windows, Version 20.0, Armonk, NY: IBM Corp.). Differences were assessed to be significant at p ≤ 0.05. Information about results always refers to this time range, unless otherwise stated. Results obtained after day 6 were not statistically evaluated due to small sample numbers.

## Results

### Comparison of clinical and biochemical alterations in piglets infected with STEC O104:H4 and STEC O157:H7

While animals in the control group stayed healthy, all animals infected with *E. coli* O104:H4 showed a transient reduction of milk intake. Four pigs out of this group became dehydrated due to diarrhoea starting at day 4 after infection. Two animals developed mild neurological disorders between days 2–6 after infection. Three pigs infected with *E.* *coli* O157:H7 developed severe clinical signs and had to be euthanized on days 4 and 5 according to preassigned termination criteria. In two of these pigs, neurological disorders were determined, which were characterised by swaying, incoordination, head shaking and excitation. The third animal was severely dehydrated due to liquid diarrhoea. One animal infected with *E. coli* O157:H7 survived until the end of trial (day 11 p.i.) but was continuously dehydrated due to diarrhoea. In infected animals, the mean average daily weight gain (ADWG) was 98 g (*E.* *coli* O104:H4 infected animals) and 74 g (*E.* *coli* O157:H7). ADWG of *E. coli* O157:H7 infected animals was significantly lower than the ADWG in control animals (115 g, p = 0.034). During necropsy liquid intestinal content was found in the gut of all infected animals. Clinical data are summarized in Table 1.

No differences between groups regarding haematocrit, haemoglobin levels as well as erythrocyte and thrombocyte numbers were found (Table 4). A higher percentage of normoblasts was found early after infection in *E.* *coli* O157:H7 infected animals in comparison to control pigs (p = 0.032) and *E. coli* O104:H4 infected pigs (p = 0.010). One animal infected with *E. coli* O157:H7 showed thrombocytopenia on day 5 after infection (215 × 10^9^ thrombocytes/l).

**Table 4:** Laboratory diagnostic findings during the course of infection in blood

		Negative			*E.* *coli* O104:H4			*E.* *coli* O157:H7		
		Median (min–max); number of animal examined			Median (min–max); number of animal examined			Median (min–max); number of animal examined		
	Time range: reference range	T1	T2	T3	T1	T2	T3	T1	T2	T3
Red blood count										
Haematocrit L/l	0.21–0.37	0.29 (0.24–0.34); 3	0.24 (0.19–0.27); 3	(0.24–0.29); 2	0.25 (0.19–0.35); 6	0.23 (0.21–0.29); 6	0.26 (0.18–0.33); 5	0.22 (0.14–0.31); 4	0.26 (0.23–0.33); 3	0.3; 1
Haemoglobin g/l	75.0–138.9	95.0 (78.0–112.0); 3	95.9 (79.3–124.0); 3	(72.5–93.8); 2	81.5 (64.0–121.0); 6	87.3 (69.9–116.7); 6	83.2 (60.7–116.3); 5	72.5 (49.0–106.0); 4	78.8 (65.1–97.9); 3	96.8; 1
Erythrocytes × 10^12^/l	3.47–6.65	4.02 (3.41–5.10); 3	3.98 (2.69–4.43); 3	(4.29–4.98); 2	3.64 (2.70–5.21); 6	3.79 (3.17–4.55); 6	4.67 (2.61–5.91); 5	3.17 (2.14–4.24); 4	4.86 (3.53–5.92); 3	5.32; 1
Plateles × 10^9^/l	282–946	62 (21–107); 3	638 (543–910); 3	(571–849); 2	80 (10–229); 6	549 (358–972); 6	925 (580–1180); 5	202 (31–266); 4	723 (215–998); 3	971; 1
Normoblasts × 10^12^/l		0.09 (0.08–0.13); 3	0.08 (0.06–0.11); 3	(0.00–0.04); 2	0.06 (0.03–0.22); 6	0.05 (0.00–0.15); 6	0.22 (0.00–0.59); 5	0.09 (0.02–0.35); 4	0.33 (0.17–0.38); 3	0.93; 1
Blood chemistry										
Creatinin µmol/l	38–142	95 (84–99); 3	63 (57–67); 3	(51–55); 2	86.5 (55–100); 6	57 (53–73); 6	68 (55–78); 5	103 (86–106); 3	63 (52–72); 4	87; 1
GLDH U/l	0–11.5	1.0 (0.5–2.0); 3	(0.6–1.4); 2	n.a.	1.3 (0.6–2.1); 5	0.9 (0.1–2.1); 6	0.6 (0.5–2.0); 3	0.7 (0.3–0.9); 3	0.6 (0.2–1.0); 4	0.5; 1
LDH U/l	409–1549	2013 (1986–2830); 3	1105 (901–1445); 3	(830–1061); 2	1893 (1150–2072); 6	1309 (909–1767); 6	806 (711–1255); 5	1301 (1185–1388); 3	710 (538–804); 4	824; 1
CK U/l	43–3343	598 (509–850); 3	98 (93–144); 3	(85–195); 2	461 (292–1515); 6	194 (66–296); 6	91 (67–288); 5	303 (241–331); 3	87 (37–111); 4	96; 1
ASAT U/l	13.7–122.8	36 (36–55); 3	11 (11–22); 3	(12–20); 2	25 (20–36); 5	20 (9–29); 6	14 (11–15); 5	19 (17–26); 3	9 (7–14); 4	11; 1
Plasma protein g/l	36.65–71.71	19.9 (19.5–22.9); 3	24.8 (23.9–27.8); 3	(27.0–28.5); 2	23.9 (20.9–29.5); 5	28.8 (23.3–31.5); 6	31.2 (27.2–33.1); 5	22.7 (21.9–27.4); 3	30.1 (27.5–30.7); 4	31.4; 1
Sodium mmol/l	136.0–151.0	130.9 (129.0–132.5); 3	132.6 (130.6–133.4); 3	(129.6–134.5); 2	130.8 (125.8–138.7); 6	133.7 (133.0–137.1); 6	131.7 (130.1–135.2); 5	132.0 (129.9–134.4); 3	132.3 (129.1–135.8); 4	129.5; 1
Potassium mmol/l	3.78–6.92	4.46 (3.32–4.75); 3	6.59 (5.74–7.60); 3	(5.23–7.04); 2	4.66 (3.99–6.22); 6	5.75 (4.96–6.89); 6	5.79 (5.15–6.12); 5	4.65 (4.44–5.50); 3	5.26 (4.85–5.80); 4	4.97; 1
Acute phase proteins										
Haptoglobin mg/ml		(0.03–0.05); 2	0.02 (0.02–0.54); 3	(0.01–0.02); 2	0.04 (0.04–0.06); 3	0.39 (0.04–0.91); 6	0.27 (0.02–1.13); 5	(0.02–0.02); 2	2.62 (2.20–3.00); 4	1.27; 1
CRP mg/l		(<0.05–0.8); 3	3.0 (0.7–13.3); 3	(1.2–3.4); 2	0.7 (<0.05–7.3); 5	7.5 (1.3–203.2); 6	11.0 (<0.05–40.9); 5	(<0.05–1.7); 3	30.0 (9.5–220.1); 4	0.7; 1
LBP mg/l		2.00 (1.00–6.50); 3	2.10 (1.20–4.00); 3	(2.1–4.9); 2	2.80 (0.80–5.10); 5	4.25 (1.70–9.60); 6	4.7 (1.1–8.8); 5	1.20 (1.20–1.30); 3	5.05 (1.20–9.00); 4	7;1

*GLDH* glutamate dehydrogenase, *LDH* lactate dehydrogenase, *CK* creatine kinase, *ASAT* aspartate transaminase, *CRP* C-reactive protein, *LBP* lipopolysaccharide binding protein, *n.a.* not assessed. *T1* timerange 1, preinfectionem, *T2* timerange 2, day 4-day 6 p.i., *T3* timerange, day 7-day 12 p.i.

Electrolyte and protein concentrations as well as enzyme activities did not differ between the groups. Haptoglobin was significantly increased in *E.* *coli* O157:H7 infected pigs compared to negative controls (p = 0.032). CRP and LBP concentrations in infected animals were not significantly increased (Table 4).

Specific weights of urine and other urine parameters did not differ between the groups. All infected piglets showed viable bacteria in the native urine sediments. A selection of results is presented in Table 5.

**Table 5:** Results gained by urinalysis

Group animal number	Erythrocytes/µl (Combur 9)		Bacteria in urine sediment		Erythrocytes in urine sediment		Squamous epithelial cells		Round epithelial cells		GFR		FE water %		FE Na %	
	T2	T3	T2	T3	T2	T3	T2	T3	T2	T3	T2	T3	T2	T3	T2	T3
Negative																
1	n	n.a.	n	n	n	(+)	(+)	++	(+)	++	3.97	4.9	2.902	4.636	0.165	0.336
2	250	n.a.	n	n.a.	+	n.a.	+	n.a.	+	n.a.	4.39	4.55	3.851	n.a.	0.029	n.a.
3	n	n.a.	n	n.a.	+	n.a.	n	n.a.	(+)	n.a.	3.73	n.a.	4.682	n.a.	0.419	n.a.
*E.* *coli* O104:H4																
4	n.a.	n	n.a.	+++	n.a.	++	n.a.	+	n.a.	n	4.63	3.68	n.a.	1.945	n.a.	0.23
5	n.a.	n	n.a.	n.a.	n.a.	+	n.a.	n	n.a.	n	4.24	3.21	n.a.	2.21	n.a.	0.299
6	250	n.a.	+++	+++	n	n	++	(+)	+	n	3.42	4.03	3.796	3.611	0.034	0.841
7	n.a.	n	n.a.	++	n.a.	++	n.a.	n	n.a.	n	4.55	4.55	n.a.	3.224	n.a.	0.21
8	250	n.a.	+	n.a.	n	n.a.	n	n.a.	n	n.a.	4.72	n.a.	n.a.	n.a.	n.a.	n.a.
9	n	n.a.	+++	n.a.	+	n.a.	+	n.a.	+	n.a.	3.97	3.57	2.856	n.a.	1.061	n.a.
*E.* *coli* O157:H7																
10	50	n.a.	++	n.a.	++	n.a.	n	n.a.	n	n.a	4.1	n.a.	1.383	n.a.	0.015	n.a.
11	n.a.	250	n.a.	+++	n.a.	n	n.a.	n	n.a.	n	4.81	2.87	n.a.	2.024	n.a.	0.341
12	0	n.a.	++	n.a.	++	n.a.	n	n.a.	n	n.a.	3.85	n.a.	2.565	n.a.	0.026	n.a.
13	250	n.a.	n	n.a.	+	n.a.	n	n.a.	n	n.a.	3.47	n.a.	2.035	n.a.	0.009	n.a.

*n.a.* not assessed, *n* negative, *T1* timerange 1, preinfectionem, *T2* timerange 2, day 4-day 6 p.i., *T3* timerange, day 7-day 12 p.i.

*Escherichia coli* O157:H7 was reisolated in all infected animals in similar amounts in pure culture. *E.* *coli* O104:H4 was reisolated in four animals at all sampling times. In two pigs out of this group (ID 6 and 7) reisolation failed on the day of death. Faeces of all animals within the control group were sterile as expected. In all faeces samples from infected animals Shiga toxin ELISA gave clearly positive results. Findings resulting from bacteriological examinations and ELISA are summarized in Table 1.

### Comparison of histopathological and electron microscopical alterations in piglets infected with STEC O104:H4 and STEC O157:H7

**Fig. 2:**
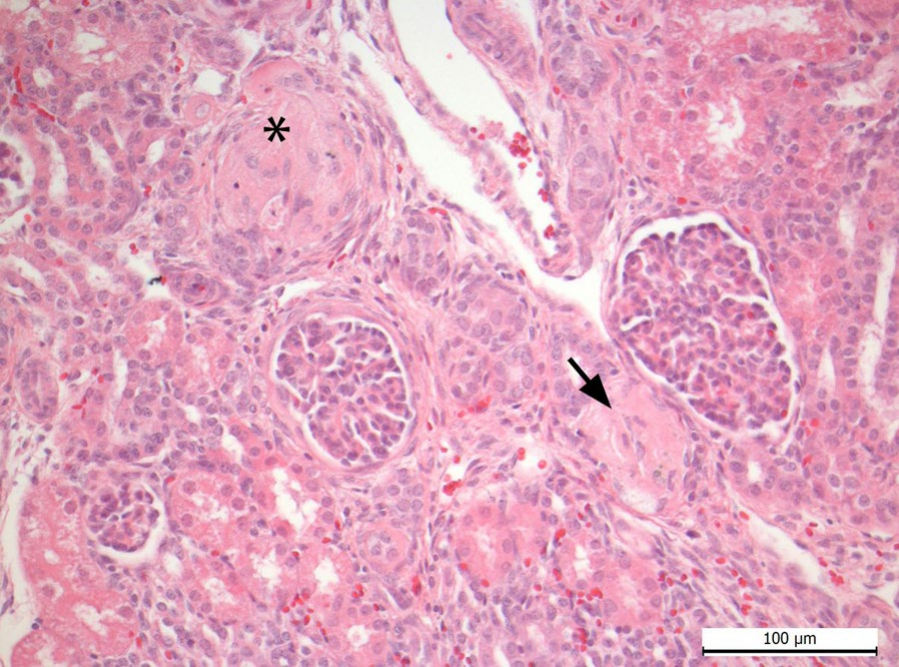
Kidney of piglet infected with *E. coli* O157:H7. Arterial thrombosis (*arrow*) and severe glomerular hyalinisation (*asterisk*) are detectable

**Fig. 3:**
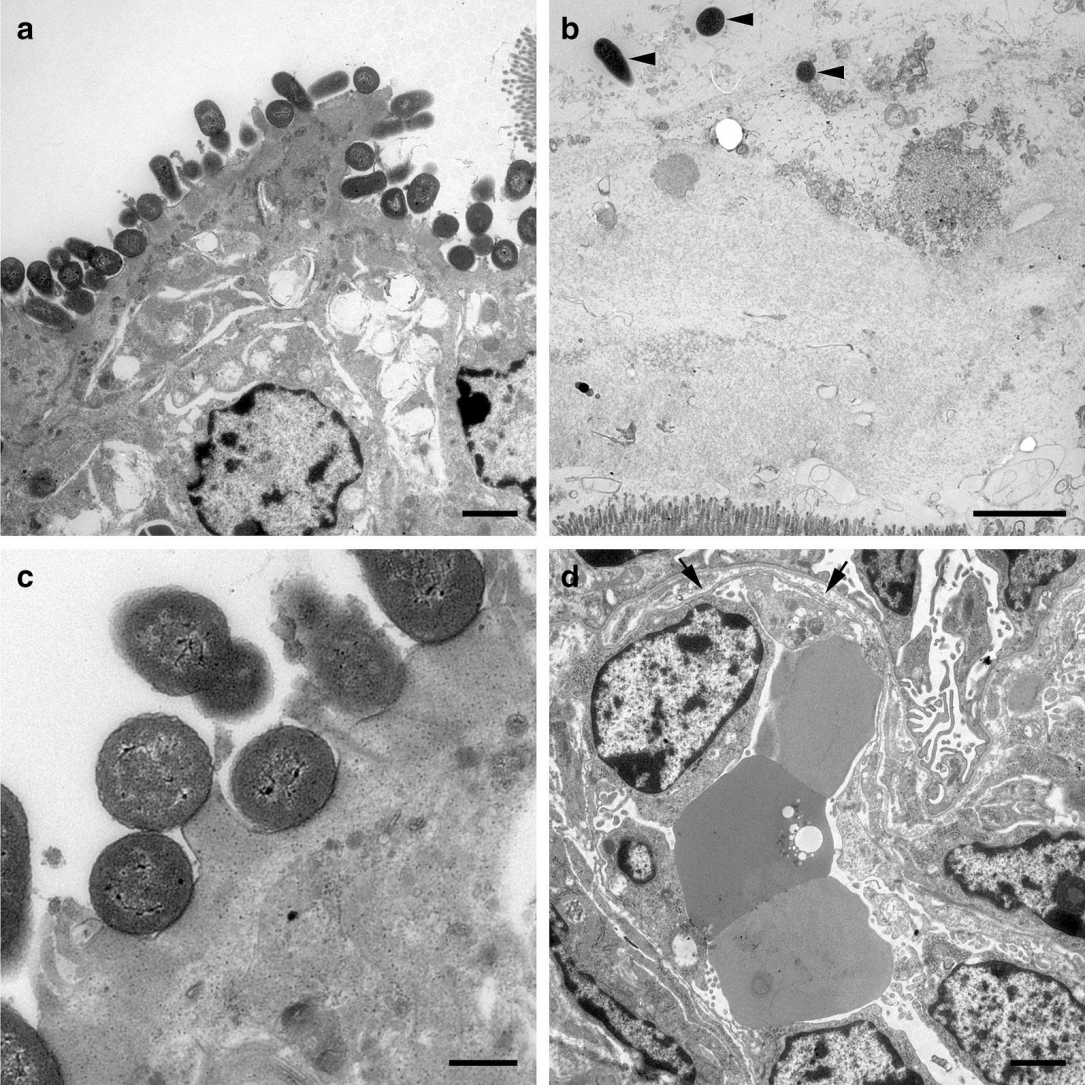
Electron microscopic images from *E. coli* O157:H7 and *E. coli* O104:H4 infected animals, respectively. **a** *Colon ascendens* of piglet infected with *E. coli* O157:H7. Bacteria are in intimate contact to epithelial cells, causing A/E lesions. *Bar* = 2500 nm. **b** *Colon ascendens* of piglet infected with *E. coli* O104:H4. Scattered bacteria in mucus above epithelial layer can be seen (*arrowheads*). No direct contact to epithelial cells is determinable. *Bar* = 2500 nm. **c** *Colon ascendens* of piglet infected with *E. coli* O157:H7. Bacteria are in intimate contact to epithelial cells, causing A/E lesions. *Bar* = 500 nm. **d** Glomerulum of the kidney of an *E. coli* O157:H7 infected piglet. Dilated subendothelial space caused by detachment of endothelial cells from basement membrane (*arrow*). *Bar* = 2500 nm

**Fig. 4:**
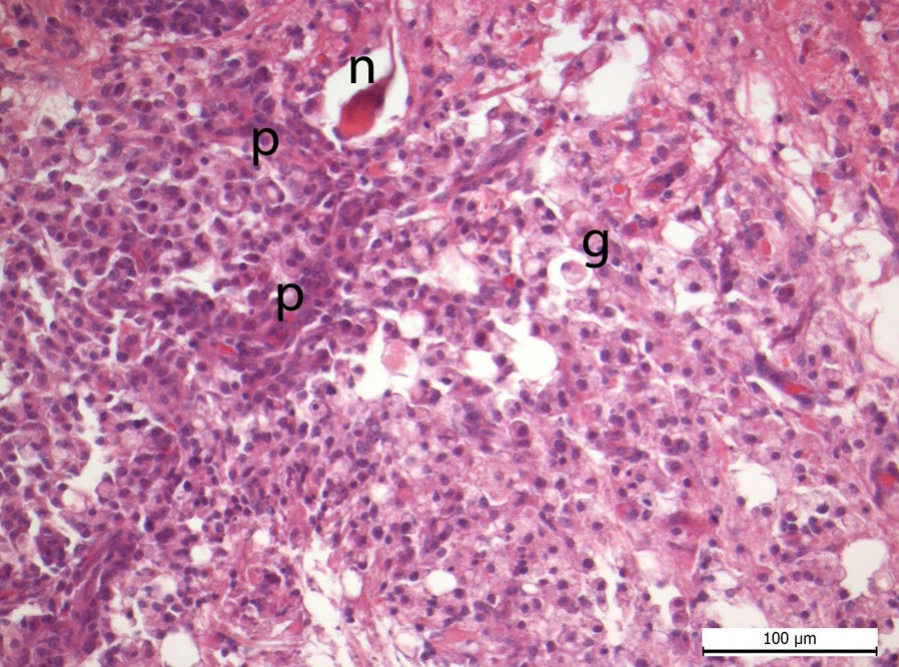
Brainstem of a piglet infected with *E. coli* O157:H7. Liquefaction necrosis: *p* proliferation of vessels, *n* neuronal necrosis, *g* gitter cells (lipid-laden microglia phagocytes)

Thrombotic microangiopathy in the kidneys was found in two pigs infected with *E.* *coli* O157:H7 (Fig. 2) and three pigs infected with *E.* *coli* O104:H4. By electron microscopy in a pig infected with *E. coli* O157:H7, dilation of the subendothelial space within the glomerula with the functional consequence of leakage was seen (Fig. 3d). One animal infected with *E. coli* 157:H7 showed acute spongiosis, another animal moderate focal liquefaction necrosis in the brainstem (Fig. 4). Histological findings from CNS, kidney and *C. ascendens* are summarized in Table 6. Results of examination of tissue samples from other organs are not shown, although minor inflammatory alterations in individual piglets were found. Electron microscopy revealed differences in the attachment of the bacteria. While in *E. coli* O157:H7 infected pigs prominent A/E lesions were found in enterocytes with pedestal formation and deprivation of microvilli (Fig. 3a, c), *E.* *coli* O104:H4 stayed in the mucus layer in distance to the epithelium (Fig. 3b).

**Table 6:** Number of animals with histopathological alterations

	Negative; n = 3	*E.* *coli* O104:H4; n = 6	*E.* *coli* O157:H7; n = 4
CNS			
Neuropil vacuolization	3	5	4
Submeningeal oedema	0	0	1
Perivascular oedema	1	5	2
Focal liquefaction necrosis in the brainstem	0	0	1
Acute white matter spongiosis in cerebellum/brainstem	0	0	1
Kidney			
Thrombosis of renal arterioles	0	3	2
*Colon ascendens*			
Submucosal oedema	3	4	3
Subserosal oedema	1	4	2
Crypt hypoplasia	0	2	0
Haematoidin/siderin/siderophages	0	2	0
Lymphoplasmocytic infiltration of the propria	0	2	1

### Immune cell subsets in blood and lymph node in piglets infected with STEC O104:H4 and STEC O157:H7

In blood, total number of leukocytes decreased in *E. coli* O157:H7 infected animals compared to the control group (p = 0.046) and *E. coli* O104:H4 infected animals (p = 0.020). Two animals infected with *E.* *coli* O157:H7 developed leukocytopenia (3.1 and 3.4 G/l, respectively; reference range 3.9–19.7 G/l; [25]). One animal infected with *E. coli* O104:H4 developed leukocytosis (20.5 G/l).

**Fig. 5:**
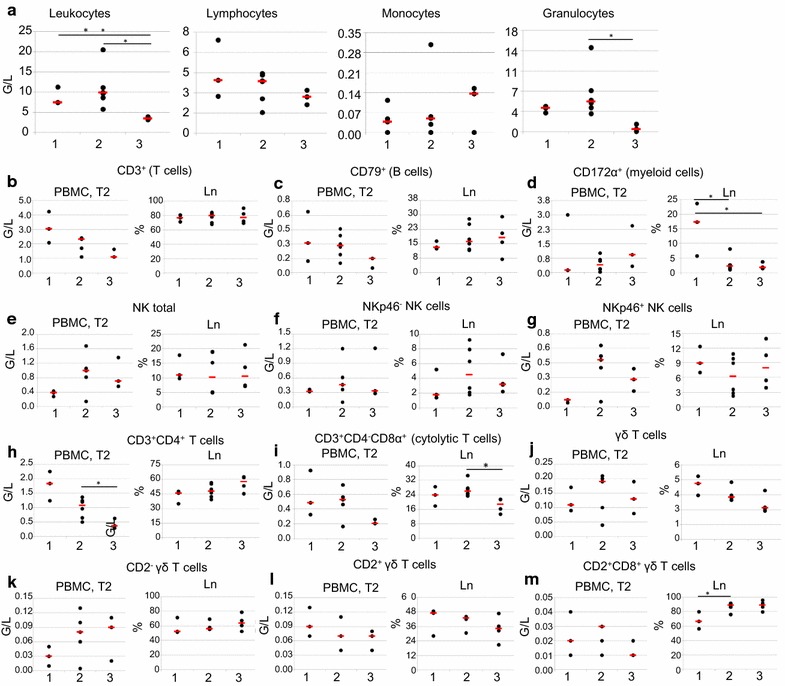
Frequencies and absolute numbers of various immune cell subsets in *E. coli* infected and control animals. *Dots* in *scatter plots* represent values of individual animals, *red lines* represent median value within group. Experimental groups are illustrated on the *x-axes*: *1* control group (n = 3), *2 E.* *coli* O104:H4-infected group (n = 6), *3 E.* *coli* O157:H7-infected group [n = 3 (PBMC), n = 4 (Ln)]. **a** Absolute number of leukocytes and leukocyte subpopulations obtained by differential counts in white blood cells from day 4 to 6 post infection. **b**–**m** Absolute number of lymphocyte subpopulations and myeloid cells in blood from day 4 to 6 post infection and percentage of lymphocyte subpopulations within live lymphocytes in lymph node (Ln) on the day of slaughter. **b** Total T cells (CD3^+^ phenotype). **c** Total B cells (CD79α^+^ phenotype). **d** Myeloid cells (CD172α^+^ phenotype). **e** Total NK cells (CD3^−^CD8α^+^ phenotype). **f** NKp46^−^ NK cells (CD3^−^CD8α^+^NKp46^−^ phenotype). **g** NKp46^+^ NK cells (CD3^−^CD8α^+^NKp46^+^ phenotype). **h** CD4^+^ T cells (CD3^+^CD4^+^ phenotype). **i** Cytolytic T cells (CD3^+^CD4^−^CD8α^+^ phenotype). [**j** Total γδ T cells (TCR-γδ^+^ phenotype]. **j** CD2^+^ γδ T cells (CD2^+^TCR-γδ^+^ phenotype). **k** CD2^−^ γδ T cells (CD2^−^TCR-γδ^+^ phenotype). **l** CD2^+^CD8α^+^CD27^+^ γδ T cells (CD2^+^CD8α^+^CD27^+^TCR-γδ^+^ phenotype)

All pigs infected with *E. coli* O157:H7 showed low numbers of polymorphonuclear neutrophils (PMN) beneath lower reference value (0.07–1.27; reference range 1.89–11.27 G/l, [25]), one animal infected with *E. coli* O104:H4 developed granulocytosis (14.56 G/l) (Fig. 5a).

For the absolute numbers of total T and B cells present in blood in tendency a decrease was found in *E. coli* infected animals compared to control animals and this decrease was more pronounced in *E. coli* O157:H7 infected pigs; somehow reflecting the drop of leukocytes counts described above (Fig. 5b, c, respectively). However, within the pool of mesenterial and ileocaecal lymph nodes no obvious change of the percentages of these two lymphocyte subsets was found between the different groups of animals. For CD172 α^+^ cells (consisting mainly of monocytes after removal of granulocytes by density gradient centrifugation) in blood very heterogeneous numbers were found across all groups (Fig. 5d). In the lymph nodes, percentages of these CD172 α^+^ myeloid cells were significantly decreased in *E. coli* O157:H7 (p = 0.034) and *E.* *coli* O104:H4 infected animals (p = 0.039) in comparison to control group, respectively.

Next to T and B cells NK cells were addressed as another major lymphocyte subset, including NKp46-defined subsets which have been investigated in pigs previously [31, 32]. Absolute numbers of NK cells and NKp46^+^ NK cells in blood were slightly enhanced in *E. coli* O157:H7 infected animals and even more in *E.* *coli* O104:H4 infected animals (Fig. 5e, g, respectively). No such changes were found for NKp46^−^ NK cells in blood (Fig. 5f) and no obvious changes were found in NK cells and related NK-cell subsets in lymph nodes (Fig. 5e–g).

In addition to total T cells (CD3^+^) the subpopulation of CD4^+^ T cells (CD3^+^CD4^+^ phenotype; representing putative T-helper cells) was investigated (Fig. 5h). Similar to the absolute number of total T cells (Fig. 5b) CD4^+^ T cells were reduced in the blood of infection groups (not significant). In tendency, a slight increase of the percentages of this population was found in the lymph nodes of *E. coli* O157:H7 infected animals (Fig. 5h). A peculiarity of porcine CD4^+^ T cells is the up-regulation of CD8α molecules on the cell surface following activation [33]. Hence, within one of the staining panels of our flow cytometry analyses monoclonal antibodies against CD8α were included (Table 2), allowing the investigation of CD8α expression in CD4^+^ T cells. However, frequencies of CD8α^+^ CD4^+^ T cells were extremely low (data not shown), which is in accordance with previously published data of newborn piglets [28]. No obvious changes in absolute counts in blood or frequencies within lymph nodes was found for this very rare T cell subpopulation between infected and control pigs (data not shown). Within the T cell subpopulation of CD3^+^CD4^−^, CD8α^+^ cells (representing cytolytic T cells) were analysed (Fig. 5i). In blood of *E. coli* O157:H7 infected animals total numbers of cytolytic T cells were decreased compared to control pigs and *E.* *coli* O104:H4 infected animals. But these differences were not significant. In lymph nodes, percentage of this population was significantly decreased in *E. coli* O157:H7 infected animals compared to animals infected with *E.coli* O104:H4 (p = 0.011). γδ T cells are another prominent T cell subpopulation in the blood [34] and secondary lymphatic organs of pigs [35]. For absolute numbers of γδ T cells in blood, a substantial variation among different piglets was found but without an obvious link to *E. coli* infection (Fig. 5j). Within lymph nodes percentages of γδ T cells were slightly, but not significantly reduced in infection groups. Expression of the cell surface molecules CD2, CD8α and CD27 could be recently related to different stages of γδ T cell development. For CD2 it was shown that already in the thymus two different lineages of γδ T cells can be identified by this marker [36]. For CD8α and CD27 published data suggest that within CD2^+^ γδ T cells a CD8α^+^CD27^−^ phenotype is related to a late stage of differentiation [35]. Hence, we analysed expression of CD2, CD8α and CD27 in γδ T cells also in this study (Fig. 5k–m). Absolute numbers in blood for CD2^−^ γδ T cells were slightly increased in *E. coli*-infected groups (Fig. 5k), whereas CD2^+^ γδ T cells were slightly reduced (Fig. 5l). No clear infection-related changes were found in the CD2-defined γδ T cell subsets analysed in lymph nodes (Fig. 5j, k). When CD2^+^ γδ T cells were analysed for CD8α and CD27 expression, the vast majority of cells had a CD8α^+^CD27^+^ phenotype (Fig. 5l, m). The absolute numbers of this γδ T cell subset in blood showed no infection-related changes, whereas the percentages of CD8α^+^CD27^+^ γδ T cells within CD2^+^ γδ T cells in lymph nodes was slightly enhanced in infection groups.

### Cytokine/chemokine response in piglets infected with STEC O104:H4 and STEC O157:H7

Cytokine/chemokine responses are summarised in Table 7. IFNγ was significantly increased in both infection groups compared to healthy controls (p = 0.038 in *E. coli* O104:H4 and p = 0.031 in *E. coli* O157:H7 infected animals, respectively). IL8 in measurable amounts (34.2 pg/ml) could only be found in a single animal infected with *E. coli* O157:H7 on day 11 after infection (data not shown). IL1β could be measured at very low and comparable levels in all groups. No IL4 response could be measured in any animal (data not shown). IL12 levels were comparably low in all animals, with the exemption of one animal in each infection group (714.l8 pg/ml in an *E. coli* O104:H4 infected animal and 6247 pg/ml in an *E. coli* O157:H7 infected animal, respectively). A decrease in CCL2 could be assessed in *E. coli* O157:H7 infected animals in comparison to the control group (p = 0.034). IL10 was significantly increased in piglets infected with *E. coli* O104:H4 (p = 0.020) and not significantly increased in *E. coli* O157:H7 infected animals.

**Table 7:** Systemic cytokines/chemokines measured in serum of piglets during course of infection using FMIA

	Negative			*E.* *coli* O104:H4			*E.* *coli* O157:H7		
	Median (min–max)			Median (min–max)			Median (min–max)		
Time range:	T1, n = 3	T2; n = 3	T3; n = 2	T1; n = 5	T2; n = 6	T3; n = 5	T1; n = 3	T2; n = 4	T3; n = 1
IL1β pg/ml	6.3 (6.3–14.6)	4.7 (3.3–6.5)	5.6 (5.6–5.7)	5.6 (5.0–8.1)	5.6 (4.6–8.4)	5.9 (3.9–7.0)	7.7 (6.7–8.4)	7.5 (5.5–8.9)	8.8
IL12 pg/ml	279.7 (195.0–413.9)	104.0 (101.4–126.9)	2263.4 (106.5–4420.4)	194.9 (152.8–10856.4)	165.9 (91.3–714.8)	91.3 (42.4–139.8)	134.7 (126.9–243.1)	197.1 (93.9–6247.2)	352.5
CCL2 pg/ml	807.1 (730.7–3973.4)	1529.7 (840.6–2935.0)	2550.6 (1751.2–3350.0)	536.4 (251.5–1176.4)	1372.8 (570.1–3598.0)	1917.8 (826.3–4011.7)	603.1 (441.9–672.1)	453.9 (365.4–513.5)	677.3
IFNγ pg/ml	2.8 (0.0–13.6)	0.0 (0.0–16.3)	4.0 (0.0–8.0)	0.0 (0.0–4.2)	38.7 (2.8–582.8)	5.5 (0.0–63.6)	0.0 (0.0–4.2)	48.3 (46.2–98.3)	30.5
IL10 pg/ml	49.4 (45.0–51.5)	11.2 (9.2–15.4)	17.1 (16.7–17.5)	29.5 (22.0–33.1)	20.1 (18.8–30.0)	11.5 (9.8–17.5)	22.7 (22.5–26.2)	19.4 (14.6–31.4)	26.7

*T1* timerange 1, preinfectionem, *T2* timerange 2, day 4–day 6 p.i., *T3* timerange, day 7–day 12 p.i.

## Discussion

In this gnotobiotic piglet infection experiment with German LEE-negative *E. coli* O104:H4 outbreak strain and LEE-positive *E. coli* O157:H7 main parameters indicative for STEC related disease in humans were analysed. Differences in clinical outcome between the two infection groups were obvious. While three out of four *E. coli* O157:H7 infected animals developed severe disease and had to be euthanized within 5 days after infection, animals infected with *E. coli* O104:H4 developed only mild signs of disease with diarrhoea and dehydration as the most consistent clinical signs in this group. In human patients, incubation time of STEC is described to be between 2 and 10 days, with a latency between occurrence of gastrointestinal symptoms and the onset of HUS of approximately 7 days [37, 38]. In the STEC O104:H4 outbreak that struck Germany in 2011 the average incubation time was 8 days with a latency from onset of gastrointestinal symptoms of HUS of approximately 5 days [2]. Thus, it cannot be ruled out that *E.* *coli* O104:H4 infected animals in this study would have developed signs of systemic disease, if kept alive longer.

However, due to the restricted space in the isolator units the duration of experiments was limited which was one drawback using this gnotobiotic piglet model. Further drawbacks were the low sample size and that the piglets´ morphological and immunological status of the gut differs from a physiological situation. In the immature gut of neonate piglets infiltration of T-cells and antigen-presenting cells occurs within days after birth, but maturity of the gut is reached not before the sixth week of age [39, 40]. In germ-free animals in general the development of the mucosal immune system is hampered by lack of a physiological colonization with bacterial microflora around birth [39, 40]. This restriction cannot be overcome in the artificial rearing system, which is the main reason for using this model to study systemic responses towards a single pathogenic stimulus. Cell culture models are advantageous for the analysis of specific pathomechanisms on a cellular level. In a model with human IECs the shedding, content and uptake of membrane vesicles of *E. coli* O104:H4 was demonstrated. In this model a Gb3 and Stx2a-independent mechanism for Stx2a uptake and important pathomechanisms, namely apoptosis of cells by caspase-9 and caspase-3 activation as well as induction of IL8 secretion were shown [6]. Animal models with all their restrictions and cell culture models deliver complementary data. In this gnotobiotic piglet model we found differences in the levels of systemic early immune reactions in combination with clinical, pathological and bacteriological findings.

Bacterial loads in faeces could be evaluated on different days after infection. It was found that both STEC strains colonised the intestine without significant differences in colony counts. Re-isolation failed on the day of necropsy in two animals infected with *E. coli* O104:H4, but PCR-analysis was positive, so that bacteria might have been killed during sample processing. The question, if the amount of Stx produced in the gut and transferred to the blood stream is different between the strains cannot be answered in this model, but Stx production of both strains in vivo could be confirmed by stool analysis. A method for quantitative determination of Stx2-serum levels is not available so far. The question, why human infections with *E. coli* O104:H4 in 2011 led to severe clinical outcomes with a high percentage of patients developing HUS and an epidemic course of disease, remains unclear. Disease in piglets caused by this STEC strain did not resemble reported cases in humans, because systemic disease could not be induced. Gnotobiotic piglets seem to be more susceptible to *E. coli* O157:H7 than to *E. coli* O104:H4 related disease.

In electron microscopy the different ways of adherence of the tested strains were visible. It can be hypothesised that the mild clinical symptoms in *E. coli* O104:H4 infected pigs can be attributed to the missing intimate adherence of the strain to enterocytes.

Data about experimentally induced thrombotic microangiopathy (TMA) in piglets infected with STEC are inconsistent. While Francis et al. [41] did not find glomerular damage in *E.* *coli* O157:H7 infected piglets, Gunzer et al. [15] found TMA in 5 out of 6 animals infected with *E.* *coli* O157:H7 and four animals infected with *E. coli* O26:H11. In the present trial we could confirm the findings of Gunzer et al. [15]. TMA was present in kidneys from 2 out of 4 piglets infected with *E.* *coli* O157:H7 and in 3 out of 6 animals infected with *E.* *coli* O104:H4. In a pig infected with *E.* *coli* O157:H7 kidney alterations were identified by electron microscopy revealing dilations of subendothelial spaces within glomerula resulting in leakage. Due to the lack of uraemia in the infected piglets, kidney alterations were assessed to be not of impact for organ function. However, other laboratory findings characteristic for HUS in humans could not be observed. In human HUS patients increased numbers of PMN are a negative prognostic marker for the outcome of disease and correlate with severe clinical signs [42]. Granulocytosis could be reproduced in a murine infection model and absence of elevated PMN numbers was correlated to a better clinical outcome [43]. This is in contrast to the findings in this study, as piglets infected with *E.* *coli* O157:H7 showed a tendency of lower numbers of PMN and a decrease in total leukocyte cell counts in comparison to the other two groups. The numbers of PMN in pigs infected with *E.* *coli* O104:H4 were only slightly increased compared to the control group. The absence of specific alteration of blood parameters indicative for HUS is in accordance to the findings of Gunzer et al. [15]. It can be hypothesised that typical alterations of blood counts do not develop in *E. coli* O157:H7 infected pigs because of the rapid onset of disease.

Thrombocytopenia can be considered as one of the earliest signs of HUS [44], although not in all human cases thrombocytopenia was observed. Thrombotic microangiopathy without a decrease in thrombocyte numbers was found frequently in humans [45, 46]. Neither in the present study nor in previous studies thrombocytopenia was reproduced in gnotobiotic piglets orally infected with STEC [15]. Age of animals and blood losses after Caesarean section might contribute to the low thrombocyte numbers on the first day of life.

An increase of the enzyme lactate dehydrogenase (LDH) can be observed in most human HUS patients [45]. Neither in this experiment nor in previous trials activities of LDH or other enzymes like glutamate dehydrogenase (GLDH) aspartate transaminase (ASAT), or creatine kinase (CK) were consistently increased in infected pigs [15]. A decrease of the acute phase protein haptoglobin reflects haemolysis, while an increase is indicative for inflammation. In this study haptoglobin concentrations were significantly increased in *E.* *coli* O157:H7 infected animals. This can be interpreted as an early sign of inflammation with or without haemolysis. High concentrations of CRP were predictors for neurological complications in *E.* *coli* O157:H7 infected human patients [47] and renal insufficiency in persons with TMA [48]. The observed higher concentrations of CRP in individual infected animals are therefore in accordance with literature. One out of three animals with increased CRP concentrations after infection developed neurological disorders. A lack of knowledge in cellular immune reactions during STEC infections and the recent findings about a decisive role of NKT cells for Stx related pathological alterations [11] was initiative for studying different cell populations in different tissues by flow cytometry. In swine NKT cells as defined for mice and humans have not been undoubtedly identified so far, but there are first indications that T cells with NK cell characteristics exist also in swine [49]. NK cells are considered as the first line of defense already in new born animals [28].

No major changes could be found for most of the analysed lymphocyte subpopulations and myeloid cells. Several major lymphocyte subsets in blood followed the observed leukopenia in *E.* *coli* O157:H7 infected pigs, namely total (CD79α^+^) B cells, total (CD3^+^) T cells and CD4^+^ (CD3^+^) T cells. Within mesenterial and ileocaecal lymph nodes, which we assumed to be of relevance due to their anatomical proximity to the gut, only CD172α^+^ myeloid cells showed a significant reduction in both groups of *E. coli* infected pigs. The reasons for this are speculative but may indicate a general influx of a mixture of other lymphocytes, since no obvious increase of other investigated lymphocyte subsets was observed. Of note, for CD4^+^ T cells which are frequently involved in immune responses to bacterial infections, for example via IL17 production [50, 51], no up-regulation of the activation-associated CD8α [33] molecule could be found. This may indicate that the cellular immune system of these newborn piglets is still in a very immature state. In contrast, important immunological effector cells in newborn piglets were the NK cells, which had been found to be perforin positive already at the day of birth, so that an immediate cytotoxic activity of these cells is guaranteed. It was shown previously in piglets that already at birth NKp46 expression on NK cells differed between individuals and that no correlation with environmental factors or age exists [28]. It could not be answered from the results of this study, if NK cells are involved in pathogenesis during STEC infection as shown for NKT cells in mice [11], or if porcine NK cells have a protective effect.

To further address immune responses in STEC infected piglets, we performed FMIA using serum samples. Different cytokines/chemokines were previously determined as parameters for inflammation in HUS-patients in different studies. According to data from the literature we considered levels of cytokines IL1β and IL10 and chemokine IL8 to be of greatest interest in STEC-infection. While IL8 was elevated in D + HUS-patients in comparison to patients with viral or bacterial gastroenteritis, IL10 levels of HUS-patients were decreased [52]. Increased concentrations of IL8 as well as of IL1β in children suffering from acute HUS could also be shown by Inward et al. [53]. In contrast, Litalien et al. [54] found elevated levels of IL10 and unaltered IL8 levels in HUS-patients in comparison to healthy controls. This is in accordance with our findings of an IL10 increase in infected animals and no detectable differences in IL1β or IL8 response between groups. The significantly increased levels of IFNγ in infected groups may be related to the observed higher number of NKp46^+^ NK cells in blood of these animals. In swine, it has been shown, that NKp46^+^ NK cells produce more IFNγ than NKp46^−^ NK cells [31].

CCL2, also known as monocyte chemoattractant protein-1 (MCP-1), is assumed to play a role in pathogenesis of HUS by recruitment of monocytes and PMNs to the kidneys [55]. A local role of this chemokine in the urinary tract could not be addressed in our study. Serum concentrations of CCL2 were significantly decreased in piglets infected with *E. coli* O157:H7.

It seems, that the role of IL4 in swine is different than in mice and humans [56]. Therefore, absence of IL4 response in our study might not reflect the situation in human STEC infections. IL12, which is a link between innate and adaptive immune system and drives immune responses to a T helper 1 response [57] was elevated only in two infected animals.

As bacteriaemia caused by STEC seems to be a rare event and has only been reported in a few cases so far [58, 59], systemic immune response in human patients or infected animals may differ from results gained by cell culture experiments. Thus, divergent results from cytokine/chemokine analyses following exposure to Stx or EHEC in various studies may be explained—at least in part—by different experimental conditions in vivo and ex vivo, and by the kind of biomaterials analysed.

Immunological findings in piglets reported here reveal parameters, which should be further investigated in humans to elucidate pathomechanisms triggered by the hosts’ immune defense during STEC induced disease. In general, the complex data set obtained from animal models can indicate requirements for in-depth systematic analysis of specific mechanisms in vitro. Hence, animal and in vitro models are two options delivering complementary data and should be further exercised.

## Conclusions

In this gnotobiotic piglet model, classical EHEC O157:H7 as well as emerging STEC O104:H4 both successfully colonised in the gastrointestinal tract of gnotobiotic piglets. However, differences in the way of bacterial adhesion could be observed in vivo. No systemic disease could be induced by infection with *E. coli* O104:H4, so that piglets seem to be less susceptible to this pathogen than to *E. coli* O157:H7, which is in contrast to humans. Immunological findings might suggest the importance of NK cells as early immune effector cells in newborn piglets. Thus, it should be further elucidated, if this cell population plays a key role in HUS pathogenesis.

## Abbreviations

A/E: attaching and effacing
ADWG: average daily weight gain
ASAT: aspartate transaminase
CBA: Colombia blood agar
CCL: chemokine ligand
CFU: colony forming unit
CK: creatine kinase
CNS: central nervous system
CRP: C-reactive protein
D + HUS: diarrhoea-positive haemolytic uraemic syndrome
*E. coli*: *Escherichia coli*
EAEC: enteroaggregative *Escherichia coli*
EHEC: enterohemorrhagic *Escherichia coli*
EPEC: enteropathogenic *Escherichia coli*
ESBL: extended-spectrum beta-lactamase
FMIA: fluorescent microsphere immunoassays
FSC/SSC: forward scatter/side scatter properties
Gb3: glycosphingolipid receptor globotriaosylceramide
GLDH: glutamate dehydrogenase
HE: haematoxylin–eosin
HUSEC: *Escherichia coli* causing haemolytic uraemic syndrome
IEC: intestinal epithelial cells
IL: interleukin
IL1β: IL-1-beta
IFNγ: interferon gamma
LBP: lipopolysaccharide binding protein
LDH: lactate dehydrogenase
LEE: locus of enterocyte effacement
LPS: lipopolysaccharide
NaCl: sodium chloride
NK cells: natural killer cells
NKT: natural killer T cells
OD: optical density
OMV: outer membrane vesicles
MCP1: monocyte chemoattractant protein-1
PBMC: peripheral blood mononuclear cells
PBS: phosphate-buffered saline
PMN: polymorphonuclear neutrophils
SMAC: sorbitol MacConkey agar
STEC: Shiga toxin producing *Escherichia coli*
Stx: Shiga toxin
